# Massively parallel gene expression variation measurement of a synonymous codon library

**DOI:** 10.1186/s12864-021-07462-z

**Published:** 2021-03-02

**Authors:** Alexander Schmitz, Fuzhong Zhang

**Affiliations:** 1 Department of Energy, Environmental and Chemical Engineering, Washington University in St. Louis, Saint Louis, MO 63130 USA; 2 Division of Biological & Biomedical Sciences, Washington University in St. Louis, Saint Louis, MO 63130 USA; 3 Institute of Materials Science & Engineering, Washington University in St. Louis, Saint Louis, MO 63130 USA

**Keywords:** Sort-seq, Protein abundance, Codon usage, Single-cell, Gene expression variation

## Abstract

**Background:**

Cell-to-cell variation in gene expression strongly affects population behavior and is key to multiple biological processes. While codon usage is known to affect ensemble gene expression, how codon usage influences variation in gene expression between single cells is not well understood.

**Results:**

Here, we used a Sort-seq based massively parallel strategy to quantify gene expression variation from a green fluorescent protein (GFP) library containing synonymous codons in *Escherichia coli*. We found that sequences containing codons with higher tRNA Adaptation Index (TAI) scores, and higher codon adaptation index (CAI) scores, have higher GFP variance. This trend is not observed for codons with high Normalized Translation Efficiency Index (nTE) scores nor from the free energy of folding of the mRNA secondary structure. GFP noise, or squared coefficient of variance (CV^2^), scales with mean protein abundance for low-abundant proteins but does not change at high mean protein abundance.

**Conclusions:**

Our results suggest that the main source of noise for high-abundance proteins is likely not originating at translation elongation. Additionally, the drastic change in mean protein abundance with small changes in protein noise seen from our library implies that codon optimization can be performed without concerning gene expression noise for biotechnology applications.

**Supplementary Information:**

The online version contains supplementary material available at 10.1186/s12864-021-07462-z.

## Background

Gene expression can vary significantly from cell to cell in an isogenic bacterial population, giving rise to phenotypic variation that affects population survival and fitness, ensemble performance, persistence, bacterial-host interaction, and probabilistic differentiation [1–5]. The underlying causes of gene expression variation are of particular importance to the fundamental understanding of cellular processes, which may enable the development of methods to control such variation, leading to more effective antibacterial treatments and more efficient bacteria-based biotechnology [6–10].

Cell-to-cell variation in protein abundance can arise from transcriptional, translational, and other processes that govern gene expression. How transcriptional processes affect the variability of gene expression between single-cells has been extensively studied [11–13]. Promoter strength, transcriptional bursting, transcription factor binding strength, as well as the copy number of RNA polymerase and mRNA degradation rate have all been shown to affect variability in mRNA copy numbers, which further affect the variability of protein abundance [14–16]. Parameters in translational processes such as mean translational rate and cell-to-cell variability in translational rate could both, in theory, contribute to variation in single-cell protein abundance [17]. Mean translational rate can be affected by multiple genetic elements, including the strength of ribosome binding sites, mRNA secondary structures, and codon usage, as well as growth-related factors such as charged tRNA concentrations and the copy number of free ribosomes [18]. These genetic elements and growth-related factors may also affect the variability of translational rate between single cells, which further influence variability of protein abundance. Due to this complexity, it is difficult to isolate how each individual parameter affects the variability of protein abundance. Codon usage, for example, has been shown to influence both translational efficiency and transcript stability, with suboptimal codons hindering translation and affecting mRNA stability [18–28]. Codon usage and bias also affect translational dynamics with low abundance tRNA isoacceptors pausing ribosomes [29] and controlling ribosomal traffic [30], particularly at the start of a gene sequence [31]. Despite significant knowledge on the effects of codon usage on mean gene expression, how and to what extent codon usage affects cell-to-cell variability in protein abundance is poorly understood. With codon optimization used as a popular method for enhancing and controlling expression [32], determining any additional consequences, such as on the variability, is important.

In this work, we constructed a library of green fluorescent protein (GFP) reporters with different synonymous codons at their 5′ coding sequence and expressed this library in *Escherichia coli* growing in defined glucose medium. We developed a high-throughput method that involves fluorescence activated cell sorting followed by sequencing (Sort-seq) [33] to analyze protein variabilities of 219 different GFP coding sequences within one experiment. Multiple methods were employed to validate the Sort-seq for high-throughput variability measurement. We found that codon usage has a large influence on the mean and variance of GFP abundance. Meanwhile, the squared coefficient of variance (CV^2^, also called noise) varies with GFP mean abundance but shows little difference for sequences with high mean protein abundance. Similar trend was also observed when analyzing variability of *E. coli* native proteins. These results illuminate the influence of codon usage to variations in protein abundance and can be potentially extended to study protein variations in other growth conditions and from other microorganisms.

## Results

### Design of a Synthetic Gene Library with synonymous codons

**Fig. 1:**
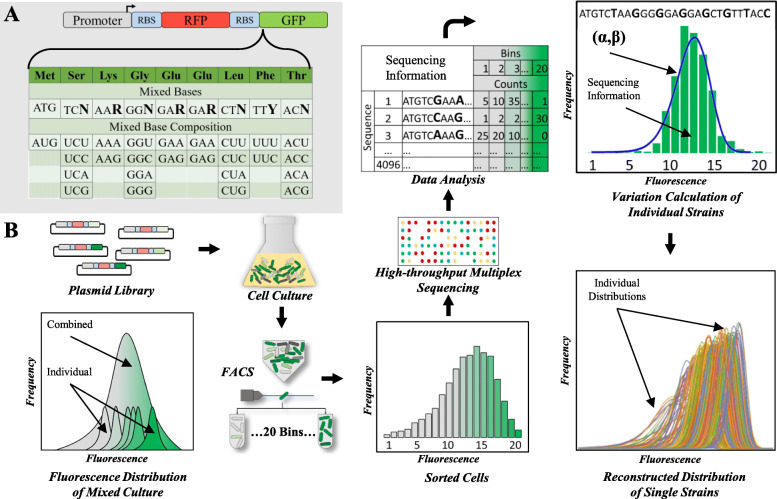
Sort-seq for massively parallel measure of protein variability. **a** Eight codons on the 5′ end of GFP are synonymously mutated. **b** Experimental procedure for massively parallel measurement of gene expression variation using Sort-seq. The plasmid library containing the synonymously mutated GFP was transformed to *E. coli* to create a pooled library. Fluorescence activated cell sorting (FACS) is used to sort the library into 20 bins based on GFP fluorescence. Plasmids are isolated from the sorted cells in each bin and are subjected to high-throughput sequencing. The number of reads for each unique GFP sequence is mapped back to each bin that represents a corresponding fluorescence intensity. Protein variability for each GFP sequence is calculated from a fitted Gamma distribution

To systematically study the influence of codon usage in cell-to-cell protein variability, a GFP library was designed with the first 8 codons after the start codon (ATG) randomly mutated to synonymous codons, resulting in a library of 4096 GFP coding sequences. All GFP coding sequences were placed to the 3′ of a red fluorescent protein (RFP) with fixed codon usage in a polycistronic structure under the control of the same promoter (Fig. 1a). RFP was used as an internal control to ensure all analyzed cells have transcribed RFP, thus eliminating cells that have lost their plasmid. The synonymous codons were placed at the N-terminal of GFP coding sequence because mean protein abundance is more sensitive to codon usage in this region due to its potential to influence translation initiation, therefore allowing us to analyze protein variabilities across a wide range of protein abundance [22]. The fluorescent reporters were expressed in *E. coli* from a low copy number plasmid (SC101 origin, approximately 5 copies) to minimize burden from gene overexpression [5].

### Sort-Seq for high throughput protein variability analysis

Protein variability was previously measured by quantifying single-cell fluorescence of a fluorescent protein using either microscopy or flow cytometry. These methods can measure variability for only one protein sequence at a time. Such low throughputs are insufficient for characterizing large reporter libraries. To solve this problem, we aimed to use Sort-seq [34] to quantify the variations of the GFP library in a massively parallel fashion (Fig. 1b). In this method, single cells are first sorted into different bins based on their GFP fluorescence. Sorted cell mixture in each bin is then sequenced using a distinctive barcode to indicate the bin. The number of reads for each unique GFP sequence is mapped to each bin that represents a corresponding fluorescence intensity. From the distribution of reads, protein variability for each GFP sequence can be calculated.

To validate the method, we first tested the number of bins that allow accurate determination of protein variability. A total of 10 testing strains from the library were randomly selected and their single-cell fluorescence distribution was measured using flow cytometry (Supplementary Figure S1). An increasing number of virtual bins were applied to each sample based on single-cell fluorescence intensity using either linear or exponential fluorescence scales to simulate the bins used in Sort-seq. The mean fluorescence for cells in each virtual bin was applied to all cells within that bin and the GFP variabilities for each strain were computed as the CV^2^_binned_. These values were compared to the variability directly calculated using the un-binned raw fluorescence distribution (CV^2^_real_). We found a consistent lower error rate when cells were binned using log-spaced fluorescence scales compared to those using linear fluorescence scales (Supplementary Figure S2) and is consistent with previous work that used log-spaced bins [35]. The percent error of CV^2^_bin_ to CV^2^_real_ also decreases as the number of bins increases (Supplementary Figure S2). With 20 bins, 8 out of 10 randomly selected strains had errors less than 5%. The other two strains with greater than 5% error at 20 bins showed less than 5% error when using fewer than 20 bins due to flow-cytometer measurement noise. Therefore, to obtain accurate quantification of protein variability, we sorted our library into 20 bins divided using an exponential fluorescence scale (Supplementary Figure S3A, S4). Compared to previous Sort-seq work for measuring mean protein abundance, a much higher number of bins are used here, reflecting the challenge in accurate quantifying of gene expression variations [34].

After sorting, plasmids from each bin were extracted, PCR-amplified using primers containing bin-specific barcodes, and sequenced. The Sort-seq experiment was performed three times to examine consistencies between experiments. A total of 5.7 million reads from 3421 unique GFP coding sequences (out of 4096 possible members in the designed library) were sequenced, representing 83% coverage of the library. For each unique GFP sequence, the number of cells distributed across different bins is calculated and fitted to a Gamma distribution based on the linearly scaled GFP fluorescence, from which mean, variance, and CV^2^ in GFP abundance was calculated (Methods). Here we calculated variabilities from a fitted Gamma distribution, instead of directly from the binned distribution, to reduce the error caused by treating fluorescence as a discrete value at each of the individual bins. The number of cells sorted per unique GFP sequence varies broadly (Supplementary Figure S3B) potentially because different GFP sequences led to different cell growth rates and thus different library member representation prior to cell sorting. We hypothesized that for sequences with too few cells-per-sequence (CPS), its variation calculation may not be accurate due to small sampling sizes. To identify the minimum CPS that provide accurate variability measurements, we grouped GFP sequences using different CPS cut-offs and compared calculated GFP fluorescence from independent Sort-seq measurements (Supplementary Figure S5). With a minimum CPS cut-off of 20, we obtain good correlation between two separate Sort-seq measurements for both mean GFP fluorescence (R^2^ > 0.94), variance (R^2^ > 0.81), and CV^2^ (R^2^ > 0.68). As the CPS cut-off drops below 20, both mean GFP fluorescence and CV^2^ correlation decrease dramatically (Supplementary Figure S5). Gating based on the CPS value excluded 92% of available GFP sequences because many GFP sequences have less than 20 cells detected. Additionally, for sequences with CPS greater than 20, we examined GFP mean and CV^2^ values measured from three independent Sort-seq experiments. GFP sequences with large percent error in either GFP mean or CV^2^ were treated as inaccurately measured and were excluded from further analysis (Supplementary Figure S6). Gating based on percent error in GFP mean and CV^2^ removed an additional 1.4% of available GFP sequences. The gating resulted in a total of 219 unique GFP sequences used in our analysis.

**Fig. 2:**
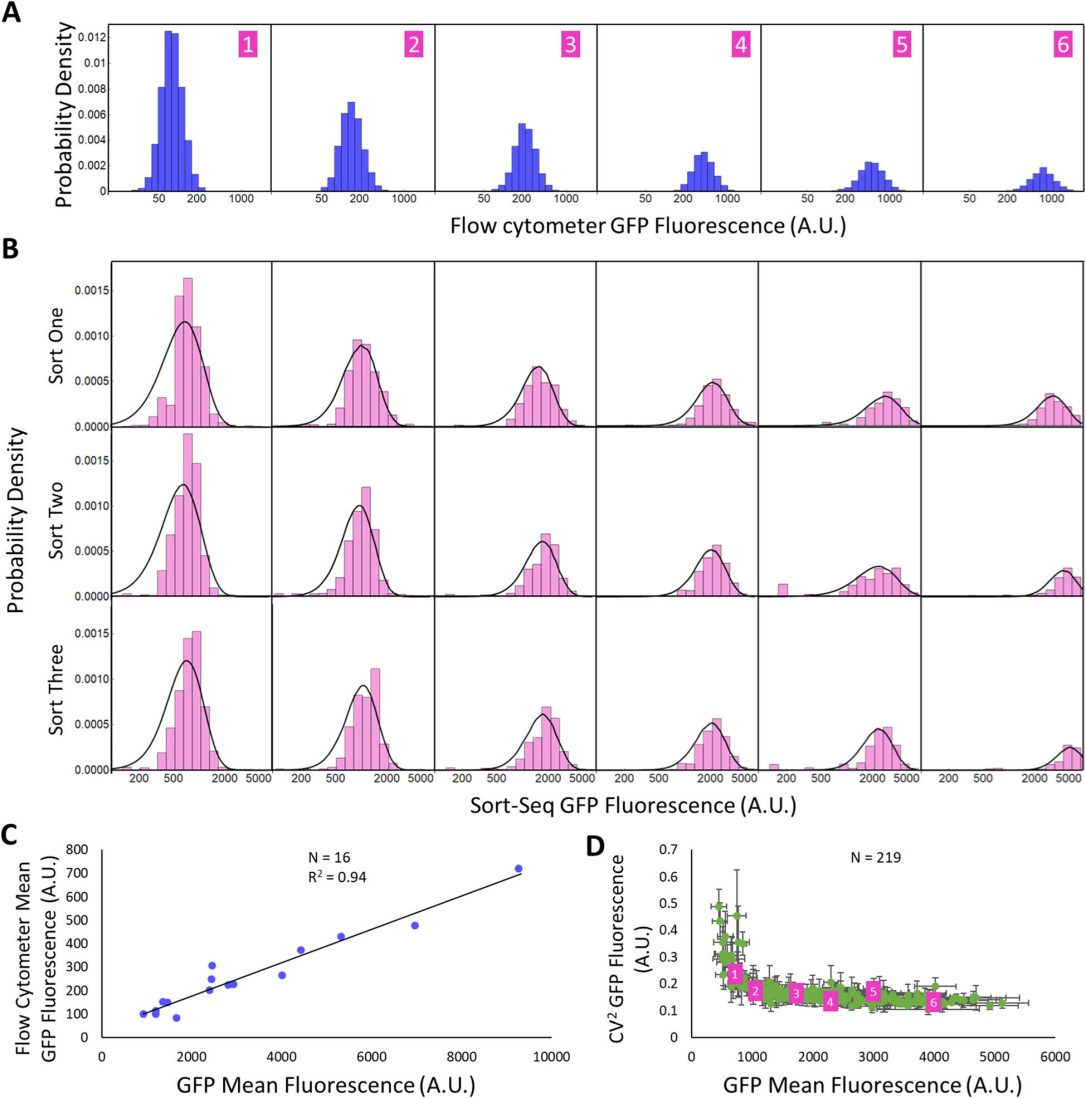
Validation of protein distribution reconstructed from Sort-seq. **a** Distributions of single-cell fluorescence as measured by flow cytometry for six randomly isolated library members. **b** Sort-seq-reconstructed single-cell fluorescence (pink columns) and the fitted curves (black) to a Gamma distribution from three independent Sort-seq experiment (from top the bottom) for the same six library isolates as shown in (**a**). **c** The correlation on mean fluorescence measured from Sort-seq and flow cytometry for another sixteen randomly isolated library members. **d** Mean GFP fluorescence and CV^2^ for all 219 library members passing our filters. Error bars represent standard deviation across the three experiments. The six isolated library members shown in (**a**) and (**b**) are highlighted in purple

The reconstructed Gamma distribution of the remaining sequences overlaps closely with Sort-seq measured fluorescence distribution across replicates (Fig. 2a and b) (Supplementary Figure S7). Additionally, we compared the mean GFP fluorescence measured from Sort-seq with those measured from flow cytometry for 16 randomly-selected individual GFP sequences which showed strong correlation (R^2^ = 0.94) for mean GFP fluorescence, further validating our method (Fig. 2c).

### Codon usage correlates with mean and variance but not CV^2^

**Fig. 3:**
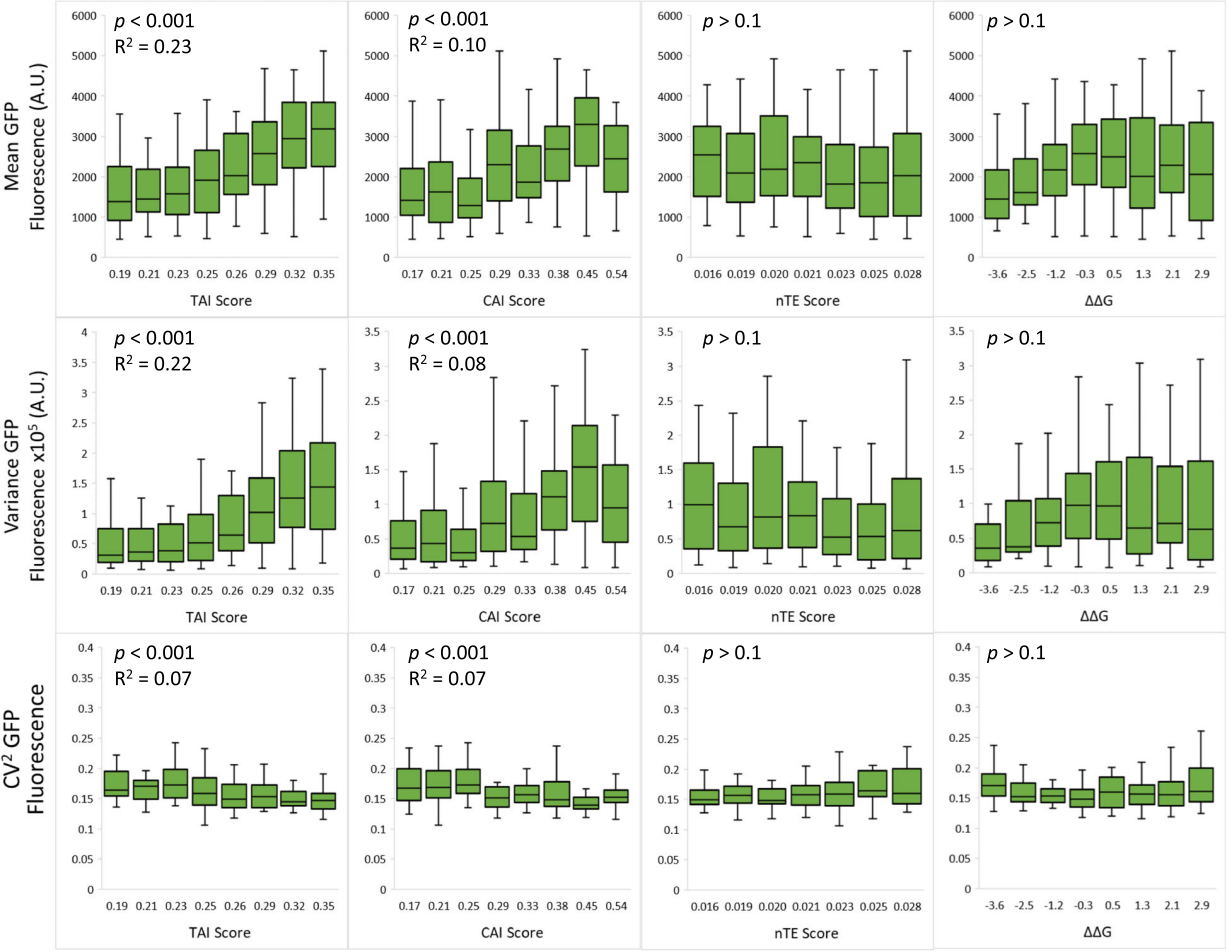
GFP fluorescence distribution parameters with various sequence metrics. GFP protein mean abundance, variance, and CV^2^ were calculated based on data measured from Sort-seq experiment. A total of (*N* = 219) library members are compared on the mean, variance and CV^2^ against sequence metrics including the TAI score, CAI score, nTE score, and free-energy difference from mean of the secondary structure of the transcript (ΔΔG). Error bars represent standard deviation

To understand how codon usage affects protein variability, GFP sequences were analyzed based on a few commonly used quantitative metrics of the 8 variable codons, including the tRNA Adaptation Index (TAI), the Codon Adaptation Index (CAI), the Normalized Translation Efficiency Index (nTE) scores and the folding free energy of the mRNA secondary structure (Fig. 3) [36–38]. The measured mean GFP abundance, variance, and CV^2^ are compared for each scored group. The mean GFP level correlates weakly with either TAI (R^2^ = 0.23, *p* < 0.001) or CAI scores (R^2^ = 0.10, *p* < 0.001), consistent with previous measurements from GFP codon libraries [39]. However, we did not observe significant correlation with the nTE score (*p* > 0.1). Because the nTE score is a measure of cellular competition for tRNAs, the lack of correlation suggests that tRNAs are likely not the rate-limiting factor for GFP translation under our experimental condition (minimal medium with 1% glucose as carbon source). We also did not observe significant correlation between mean GFP fluorescence and the folding energy of 5′ GFP mRNA (*p* > 0.1) as previously suggested [39, 40] This is potentially because GFP is the second coding sequence on the mRNA. In our construct, the GFP start codon is located 22 base pairs after the RFP stop codon, and the ribosome is known to prevent mRNA folding for a region 21 base pairs away from the ribosome A site [41]. Thus, it is likely that the folding energy of GFP mRNA is affected by ribosome translation of the 5′ RFP sequence. Similar weak positive correlations are observed between variance of GFP levels with TAI (R^2^ = 0.22, *p* < 0.001) and CAI scores (R^2^ = 0.08, *p* < 0.001), but not with the nTE score nor folding energy of 5′ GFP mRNA (*p* > 0.05). CV^2^ correlates weakly with either TAI (R^2^ = 0.07, *p* < 0.001) or CAI score (R^2^ = 0.07, *p* < 0.001), likely due to the fact that CV^2^ is large at low GFP levels (Fig. 2d).

**Fig. 4:**
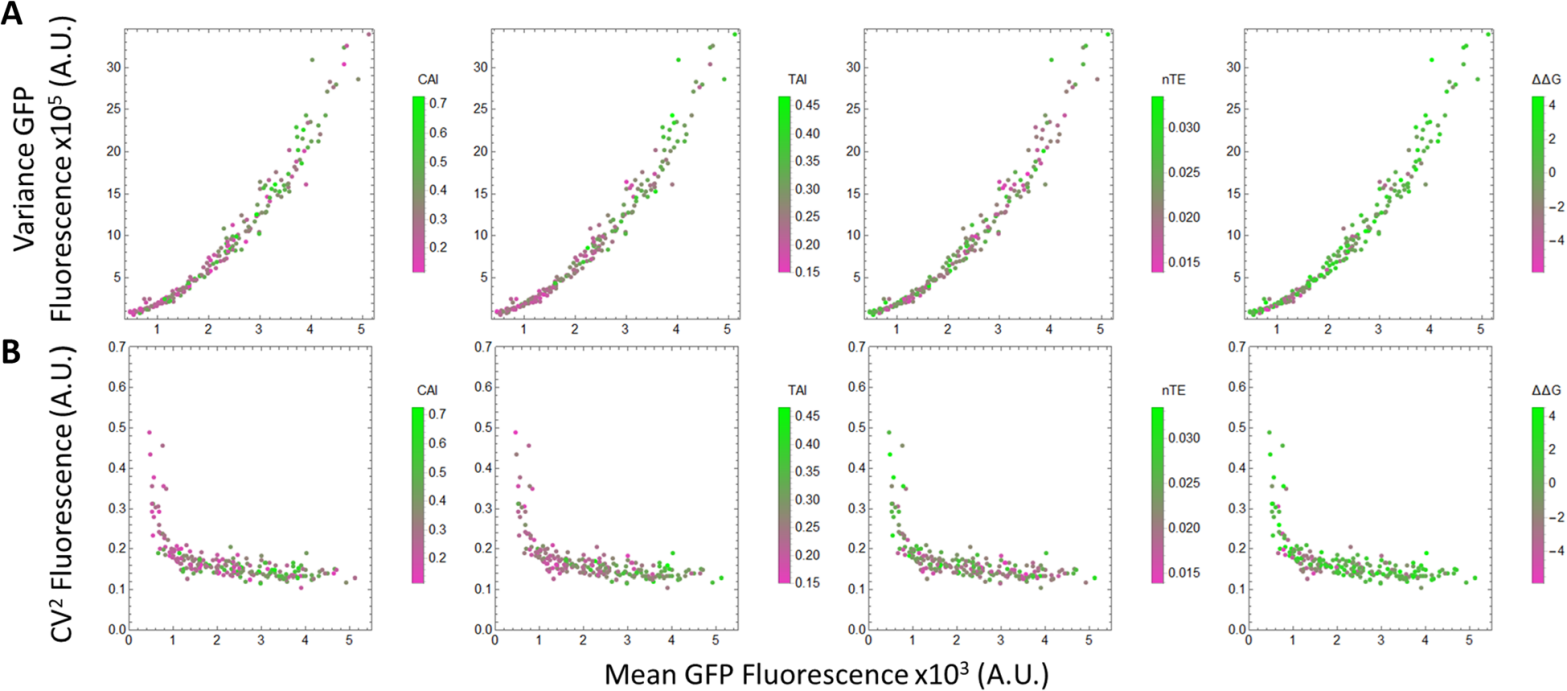
GFP Variance and CV^2^ with Mean GFP fluorescence. From the Sort-seq experiment, the variance (**a**) and CV^2^ (**b**) are compared to mean GFP protein abundance and different codon metrics including the TAI score, CAI score, nTE score and ΔΔG of the transcript for (N = 219) library members. Higher codon scores are represented by green and lower codon scores are represented by purple

Altering the codon usage has a significant effect on the mean expression level, which in turn affects variance and CV^2^. To isolate the influence of codon usage through mean expression level, GFP variance and CV^2^ are plotted against mean GFP level. While GFP variance increases with GFP mean (Fig. 4a), GFP CV^2^ generally decreases with mean at low GFP abundance and levels off at high GFP abundance (Fig. 4b), consistent with previous observations from genome-wide *E. coli* native gene expression [17]. At high GFP abundance, several sequences with the same mean displayed different CV^2^ values, but the differences are within experimental error (Fig. 2d). At high protein abundance, codon usage has little effect on protein CV^2^. Thus, codon usage affects CV^2^ mostly via affecting mean GFP level. Meanwhile, codon usage affects protein variance at all gene expression levels. Codons with high TAI or CAI scores increased both GFP mean and variance (Figs. 3 and 4).

### Codon usage Bias in the *E. coli* genome

**Fig. 5:**
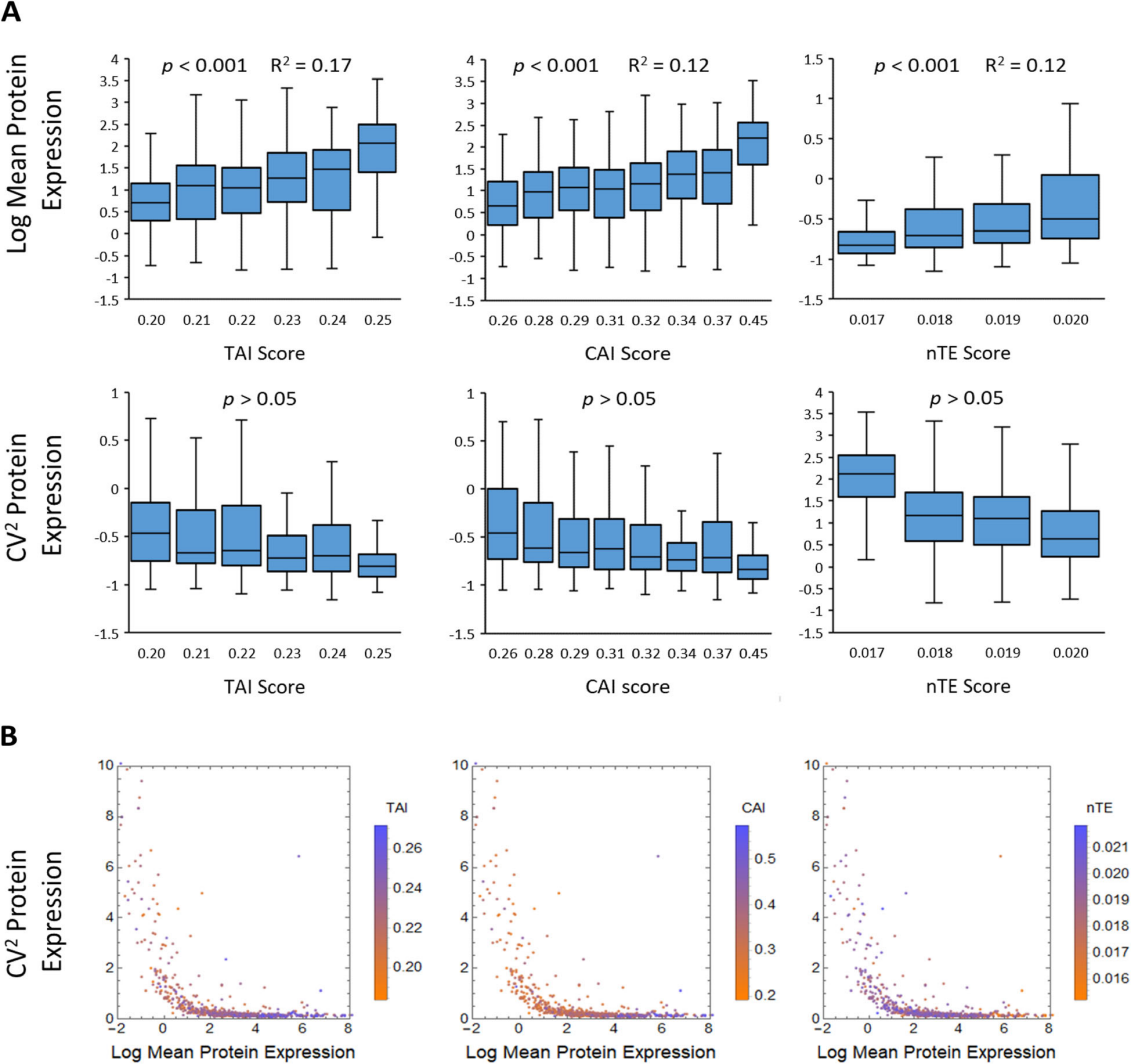
Genome analysis of codon usage for 735 genes. **a** The mean protein abundance and CV^2^ are compared to different codon metrics including the TAI score, CAI score and nTE score for (*N* = 735) native genes using all codons in the gene. **b** Protein CV^2^ compared to mean protein expression and different codon scores

In addition to testing a synonymous codon library of a synthetic gene, we also examined whether similar trends exist for native genes in the *E. coli* genome. Using protein variability of native genes measured from previous work [17], we calculated the TAI, CAI, and nTE scores of their coding sequences for 735 genes for which we had both noise information provided by a previous study [17] and sequence information provided by UniProt [42] (*E. coli* strain K-12) (Fig. 5). From the analyzed genes, weak positive correlations (*p* < 0.001) between mean expression level and TAI or CAI scores was observed, consistent with previous works [37, 38, 43]. No significant correlation (*p* > 0.05) between protein CV^2^ with any of the used codon metrics was observed (Fig. 5a). The observations from analyzing *E. coli* native genes are in agreement with results from Sort-seq analysis of our GFP library. Therefore, we conclude that codon usage only influences protein noise by affecting their mean expression levels, with little influence on highly abundant proteins.

## Discussion

The analyses performed in this study show that codon usage has a strong influence on the mean protein abundance and variance, with little influence on cell-to-cell protein variation under the same mean. The altered mean protein expression does not arise from changes in GC content (Supplementary Figure S8) or from mRNA secondary structure (Fig. 3) that could alter translation initiation. For high-abundance proteins, the lack of change in protein variability suggests that cell-to-cell variation in translational rate is not changed significantly when swapping synonymous codons. Rare codons (codons with low CAI scores) tend to decrease the mean protein abundance but only have a small effect on CV^2^. For proteins with codons requiring low-abundant tRNAs (codons with low TAI scores), their overexpression can deplete the availability of charged tRNAs. The lack of change in protein CV^2^ when swapping to codons with low TAI scores suggests that the decreased availability of tRNAs does not lead to an increase in cell-to-cell variation of charged tRNAs. This is potentially caused by the tight feedback regulation of tRNAs that would maintain tRNA levels [44]. Furthermore, our results also suggest that the main source of protein noise for high-abundance proteins is likely not translational in origin but rather due to variations in transcription, such as cell-to-cell variation in RNA polymerase as previously suggested [45].

## Conclusions

We observe that synonymously mutation of just eight codons on the GFP changed mean protein abundance by as much as five-fold (Fig. 4) with little to no change in protein noise. The drastic change in protein abundance with small changes in variation indicates that for biotechnology applications, codon optimization can be performed to control gene expression levels without concerning gene expression noise [46].

Our Sort-seq based method represents a high-throughput strategy for measuring gene expression variability. A key parameter to obtain high accuracy in variability measurement is to sort cells into a large enough number of bins to increase distribution resolution. This method can be potentially extended to other libraries, such as libraries of different promoters or RBSs, and to other organisms, illuminating genetic mechanisms that control cell variability.

## Methods

### Materials

All primers were synthesized by Integrated DNA Technologies (Coralville, IA, U.S.A.). Eco31l and T4 DNA ligase were purchased from Thermo Scientific (Waltham, MA, U.S.A.). All other reagents were purchased from Sigma Aldrich (St. Louis, MO, U.S.A). All M9 medium was supplemented with 75 mM MOPS, 2 mM MgSO_4_, 1 mg/L thiamine, 10 μM FeSO_4_, 0.1 mM CaCl_2_ and micronutrients including 3 μM (NH_4_)_6_Mo_7_O_24_, 0.4 mM boric acid, 30 μM CoCl_2_, 15 μM CuSO_4_, 80 μM MnCl_2_, and 10 μM ZnSO_4_. Plasmid DNA purification kits and fragment DNA purification kits were purchased from iNtRON Biotechnology (Seoul, South Korea). High-throughput sequencing was conducted using a MiSeq 2 × 250 standard flow cell from Illumina Inc. (San Diego, CA, U.S.A.). Sanger sequencing was conducted by Eurofins Scientific (Luxembourg). Flow-cytometry was conducted on a Guava easyCyte HT system (Luminex Corp., Austin, TX, U.S.A.) using a 488 nm laser in combination with a 525/30 filter for GFP and a 532 nm laser in combination with a 583/26 filter for RFP. Cell libraries were sorted using a BD FACS Ariall-2 cell sorter (BD Biosciences, Franklin Lakes, NJ, U.S.A.) equipped with a 488 nm laser and a 530/30 nm filter for GFP and a 561 nm laser and a 582/12 nm filter for RFP.

### Library construction

To ensure that all library members are synonymously mutated rather than randomly mutated, degenerate primers that allow specific base mutations were used to amplify a super-folder GFP (sfGFP) (Supplementary Table 1A). Both primers contain a Eco31l site for cloning purposes. Plasmid pS5c-RFP-sfGFPlibrary was constructed using one-step Golden-Gate DNA assembly [47]. The GFP library was inserted to the 3′ of a RFP coding sequence in a BglBrick plasmid pS5c-RFP [48], which contains a p15A replication origin, a chloramphenicol resistance marker, and a P_LacUV5_ promoter driving the expression of RFP. To do so, the vector backbone was PCR amplified with primers containing Eco31l sites (Supplementary Table 1B). The two PCR amplicons were digested with Eco31l, followed by ligation with T4 ligase following the Golden-Gate protocol [47]. The ligated plasmid library was then chemically transformed into *E. coli* DH10β competent cells. The transformed library was recovered in 5 mL Luria-Bertani (LB) medium for 2 h at 37 °C and then supplemented with chloramphenicol at 30 mg/mL and grown at 37 °C until reaching OD_600_ 0.08. The culture was then divided into 500 μL aliquots, mixed with 500 μL of 50% glycerol, and stored at − 80 °C until use.

### Optimizing sorting parameters

The number of bins used for the Sort-Seq protocol was determined using the flow-cytometer data from the ten individual library members. The distribution of GFP fluorescence was divided into different number of virtual bins, and the CV^2^ was calculated from the both the bins and the flow-cytometer data to determine the percent error between the two calculations (Supplementary Figure S2).

### Library sorting

Cell libraries were cultivated and treated with ice and rifampicin to halt growth and transcription and additional time was given to allow translated fluorescent protein to mature. Cells were then sorted based on both GFP and RFP fluorescence values. Gates were applied to exclude cells that did not fluoresce at the RFP channel above background, which was set using the fluorescence of wild type *E. coli* cells. Cells are only included that fluoresced RFP above the maximum RFP fluorescence of the wild type *E. coli* cells. Cells from the GFP library were sorted into 20 bins spaced based on their logarithm of GFP fluorescence. The cells were sorted for a total of eight hours during the second Sort-seq experiment, until a total of 2.16 million cells had been sorted across all 20 bins (Supplementary Figure S3). Fewer cells were sorted during the first and third Sort-seq experiments due to sorting time constraints. 269,000 cells were sorted during the first experiment and 1.89 million were sorted during the third experiment.

### High-throughput sequencing

Cells from each bin were subjected to plasmid extraction. Using plasmid DNA from each bin as templates, PCR was performed to amplify the GFP coding sequence containing the variable synonymous codons using primers containing both the Illumina Multiplex sequences (Supplementary Table 2A) with a specific index for each bin (Supplementary Table 2B). PCR was performed for 16 cycles, and the PCR products were gel purified. Purified DNA samples were combined at equal concentrations to produce a 10 nM sample that was then subjected to high-throughput sequencing using a MiSeq system 2 × 250 standard flow cell. A total of 3.9 million reads were generated on the second Sort-seq experiment.

### Examining of individual library members

To examine individual library members, 1 μL of the library aliquots was plated onto an agar-LB plate containing 30 mg/mL of chloramphenicol. From the overnight plate, 10 colonies were randomly-selected and cultivated. Their plasmid DNA were then extracted, followed by Sanger sequencing. All 10 plasmids contained the correct GFP coding sequences with non-identical synonymous codons at the expected sites. The 10 overnight cultures were also used to inoculate M9 minimal media containing 1% glycerol and 30 mg/mL chloramphenicol with a starting OD_600_ of 0.0025 and grown at 37 °C. After 2 h, the cultures were induced with 1 mM IPTG and grown until OD_600_ reached 0.08. A low OD is used to prevent clogging the flow-cytometer. Cells were then transferred to ice and incubated for 10 min followed by adding 2 μL of 50 mg/mL rifampicin to halt transcription. The culture was then moved back to 37 °C and incubated for 1 h to allow synthesized fluorescent proteins to fold and mature before flow-cytometry.

### Library quality testing

The quality of the library was confirmed by high-throughput sequencing prior to sorting to ensure proper library construction and transformation. In detail, an aliquot of the library culture was grown in 5 mL LB medium overnight. The overnight culture was then used to inoculate10 mL of minimal M9 medium containing 1% glycerol and 30 mg/mL chloramphenicol with a starting OD_600_ of 0.0025 and grown at 37 °C. After 2 h of growth, the culture was induced with 1 mM of isopropyl β-D-1-thiogalactopyranoside (IPTG). When the culture reached an OD_600_ of 0.08, cells were harvested and treated with ice and rifampicin in a similar way as described above. After sorting, the cells are subjected to plasmid extraction. The GFP coding sequence containing the variable synonymous codons was PCR amplified from the plasmid DNA mixture using primers containing both the Illumina Multiplex sequences and a unique index for each bin. The Primers used are listed in (Supplementary Table 2A), and the index used is a 9 base pair region in the forward primer and listed in (Supplementary Table 2B). Index 1 was used for the initial library confirmation. PCR was performed for 16 cycles, and PCR products were gel purified. The gel extracted DNA samples were diluted to 10 nM and subjected to high-throughput sequencing. High-throughput sequencing produced 2 million reads with 85% of reads as correct members of the library. From 2 million reads, all possible library members were observed, representing 100% coverage and validating the library construction.

### Data processing

Using the index of each read, GFP sequences were sorted into their respective bins. For each unique GFP sequence, the number of reads found in a bin was first normalized by the total number of reads in that bin. The fraction of per unique GFP sequence in each bin was then multiplied by the number of cells sorted into that bin to obtain the number of cells in each bin. GFP sequences that were distributed into less than 2 bins or with less than 20 cells per sequence (CPS) (Supplementary Figure S3B) were removed without analysis. Using all three Sort-seq experiments, error bars are calculated for each library member for both mean GFP fluorescence and CV^2^ of GFP fluorescence. Any library members with above 30% error in mean GFP fluorescence and with above 40% error CV^2^ of GFP fluorescence are excluded from further analysis. Cut-offs in percent error were determined by natural cut-offs in the distribution of the percent error (Supplementary Figure S6). Finally, a total of 219 different GFP sequences were used for protein variability analysis.

To calculate protein variability, each of the 20 bins was assigned a relative protein abundance value based on the fluorescence of each bin. The bins are sorted on their logarithm scale and so are converted to linear scale before fitting. For each GFP sequence, its distribution across 20 bins were fitted to a continuous Gamma distribution using eq. 1.

1 $$ P(x)=\frac{x^{\alpha -1}{e}^{-\frac{x}{\beta }}}{\upbeta^{\alpha}\varGamma \left(\alpha \right)} $$

where *x* is the fluorescence of each bin, α is the shape parameter and β is the scale parameter representing a gamma distribution. The mean, variance, and CV^2^ of each GFP sequence were calculated from the fitted α and β values using eqs. 2, 3, and 4 respectively.

2 $$ Mean(x)=\alpha \beta $$

3 $$ Var(x)={\alpha \beta}^2 $$

4 $$ {CV}^2(x)=\frac{\alpha {\beta}^2}{{\left(\alpha \beta \right)}^2}=\frac{1}{\alpha } $$

### Codon metrics and mRNA folding energy calculations

The CAI score for each GFP sequence was calculated from the eight variable codons using eq. (5) as described previously [36]:

5 $$ CAI(sequence)={\left({\prod}_{k=1}^L{w}_k\right)}^{1/L} $$

where *L* represents the length of the sequence in the number of codons, and *w*_*k*_ is the weight of the *k* th codon in the gene sequence. The weight for each codon was obtained from previous work [36]. The TAI score was calculated for the same region using eqs. 6, 7, and 8 as previously described [37]. Specifically, utilizing tRNA gene copy as an approximation for tRNA abundance and assuming the tRNA usage of a gene is a measure of how well that gene is adapted to the available tRNA pool. *W*_*i*_, the absolute adaptiveness value, was first calculated as:

6 $$ {W}_i={\sum}_{j=1}^{n_i}\left(1-{s}_{ij}\right){tGCN}_{ij} $$

where *n*_*i*_ is the number of tRNA isoaccceptors that recognize the *i*th codon. *tGCN*_*ij*_ is the gene copy number of the *j*th tRNA that recognizes the *i*th codon and *s*_*ij*_ is a selective constraint on the efficiency of codon-anticodon coupling as reported previously [37].

7 $$ {w}_i=\left\{\begin{array}{cc}\frac{W_i}{W_{max}}& if\ {W}_i\ne 0\\ {}{w}_{mean}& else\end{array}\right. $$

where *W*_*max*_ is the maximum *W*_*i*_ value and *w*_*mean*_ is the geometric mean of all *w*_*i*_ with *W*_*i*_ ≠ 0.

8 $$ TAI(Sequence)={\left({\prod}_{k=1}^L{w}_{ik}\right)}^{\frac{1}{L}} $$

where *L* is the length of the sequence in number of codons and *w*_*ik*_ is the weight of the *k*th codon.

The nTE score was calculated for the same region for each library member using the method described [38] and is shown in eqs. 9, 10, 11, 12 and 13:

9 $$ {U}_i={\sum}_{j=1}^g{a}_j{c}_{ij} $$

10 $$ {cu}_i=\frac{U_i}{U_{max}} $$

where *c*_*ij*_ is the sum of the counts of codon *i* in gene *j* and *a*_*j*_ is the transcript abundance of gene *j* considering all genes in genome *g*. *cu*_*i*_ is the relative estimate of how often each codon is translated in the genome.

11 $$ nTE{\prime}_i=\frac{w_i}{cu_i} $$

12 $$ {nTE}_i=\frac{nTE{\prime}_i}{nTE{\prime}_{max}} $$

13 $$ nTE(Sequence)={\left({\prod}_{k=1}^L{nTE}_{ik}\right)}^{\frac{1}{L}} $$

where *w*_*i*_ is the same as calculated by eqs. 6 and 7. *L* is the length of the sequence in number of codons and *nTE*_*ik*_ is the weight of the *k*th codon. These same methods were used to calculate the CAI, TAI and nTE for the *E. coli* genome analysis utilizing all codons in each gene (Fig. 5). The free energy of folding for the secondary structure of the transcript was calculated using *NUPACK* [40] for a region 42 base pairs before and after the codon library. The difference was calculated for each library member compared to the mean free energy of folding of all the library members analyzed. This computation is the same as previously described [39].

## Supplementary Information

**Additional file 1 **Supplementary tables and figures. **Table S1:** Primers used for cloning synonymously mutated GFP library. **Table S2:** Primers and indexes used for high-throughput sequencing. **Fig. S1:** GFP fluorescence distribution for 10 isolated library members. **Fig. S2:** Virtual binning of 10 isolated from the GFP library. **Fig. S3:** Sorting cells based on GFP fluorescence. Results for each of the bins from one of the sort-seq experiments. **Fig. S4:** Sort-seq RFP fluorescence. **Fig. S5:** Finding the minimum number of cells to use per sequence (CPS). **Fig. S6:** Percent error between three sort-seq experiments. Using all three sort-seq experiments, percent error is calculated in the measurement of both mean GFP fluorescence and CV^2^. **Fig. S7:** Sort-seq reconstructed singe cell fluorescence and the fitted curves to a Gamma distribution for six library isolates. **Fig. S8:** The GC percent content of the synonymously mutated sequence is compared to the mean and CV^2^ GFP fluorescence of each sequence.

## Data Availability

All data generated during this study are included in this published article and its supplementary information files. Datasets including sequences, squared variances, and plasmid and strain information is available at the Figshare database. https://figshare.com/s/51c4820b2bee85c94007

## Abbreviations

TAI: tRNA Adaptation Index
CAI: Codon Adaptation Index
nTE: Normalized Translation Efficiency Index
GFP: Green Fluorescent Protein
RFP: Red Fluorescent Protein
CPS: Cells-Per-Sequence
FACS: Fluorescence Activated Cell Sorting
LB: Luria-Bertani
IPTG: Isopropyl beta-D-1-thiogalactopyranoside
