# Supplementary material for: Massively parallel gene expression variation measurement of a synonymous codon library
Source: BMC Genomics. 2021 Mar 2;22:149. doi: 10.1186/s12864-021-07462-z (PMC7927243; doi:10.1186/s12864-021-07462-z)
Supplement: Supplementary file 1 — Additional file 1 Supplementary tables and figures. Table S1: Primers used for cloning synonymously mutated GFP library. Table S2: Primers and indexes used for high-throughput sequencing. Fig. S1: GFP fluorescence distribution for 10 isolated library members. Fig. S2: Virtual binning of 10 isolated from the GFP library. Fig. S3: Sorting cells based on GFP fluorescence. Results for each of the bins from one of the sort-seq experiments. Fig. S4: Sort-seq RFP fluorescence. Fig. S5: Finding the minimum number of cells to use per sequence (CPS). Fig. S6: Percent error between three sort-seq experiments. Using all three sort-seq experiments, percent error is calculated in the measurement of both mean GFP fluorescence and CV2. Fig. S7: Sort-seq reconstructed singe cell fluorescence and the fitted curves to a Gamma distribution for six library isolates. Fig. S8: The GC percent content of the synonymously mutated sequence is compared to the mean and CV2 GFP fluorescence of each sequence. [file 12864_2021_7462_MOESM1_ESM.docx]

**Supplementary Tables and Figures**

**
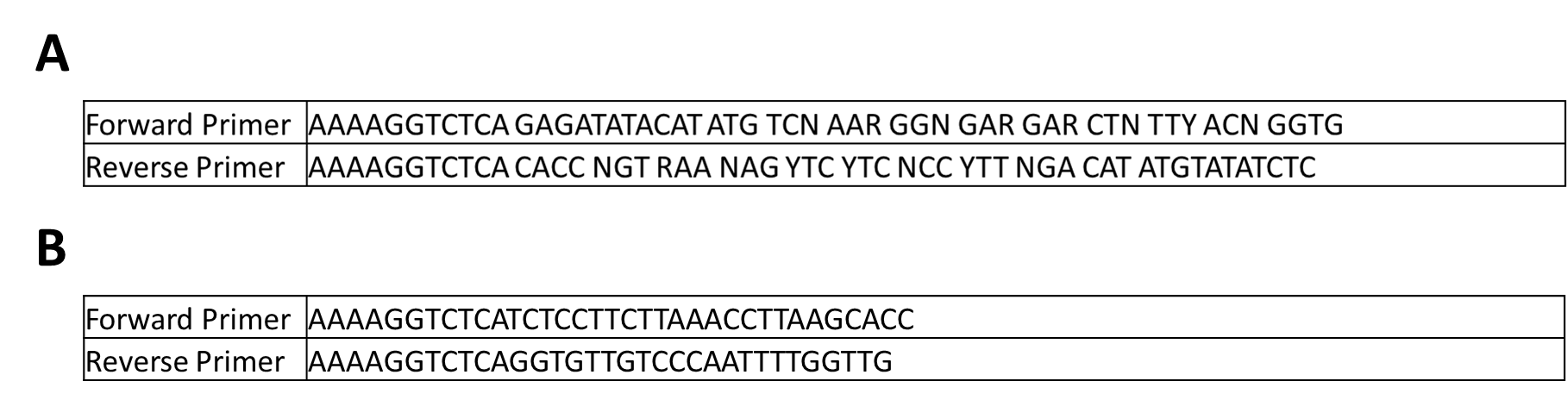
**

**Supplementary Table 1:** Primers used for cloning synonymously mutated GFP library. (A) Primers used to create the library. Mixed base pairs correspond to N = {A, C, G, T}, R = {A, G}, and Y = {C, T}. (B) Primers used to amplify the backbone from BglBrick plasmid pS5c-RFP.

**
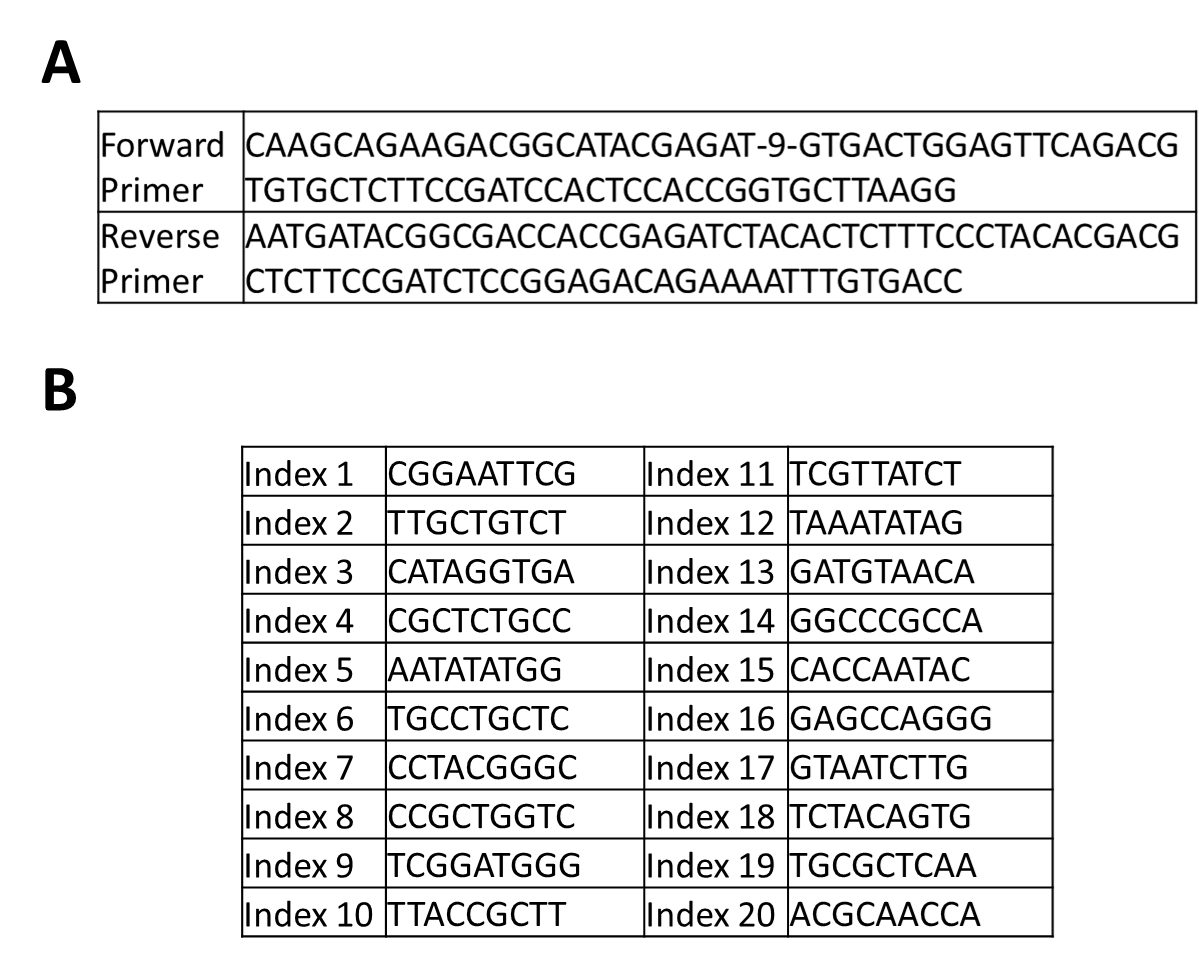
**

**Supplemental Table 2.** Primers and indexes used for high-throughput sequencing. PCR primers with Illumina adapters and indexes corresponding to each of 20 bins. (A) The forward and reverse primers used to PCR amplify the library region containing the synonymously mutated GFP. The primers contain the Illumina adapters and the 9 blank base pairs. (B) The 20 different 9 base pair indexes used for the high-throughput sequencing to identify which of the 20 bins a particular read belongs to.

**
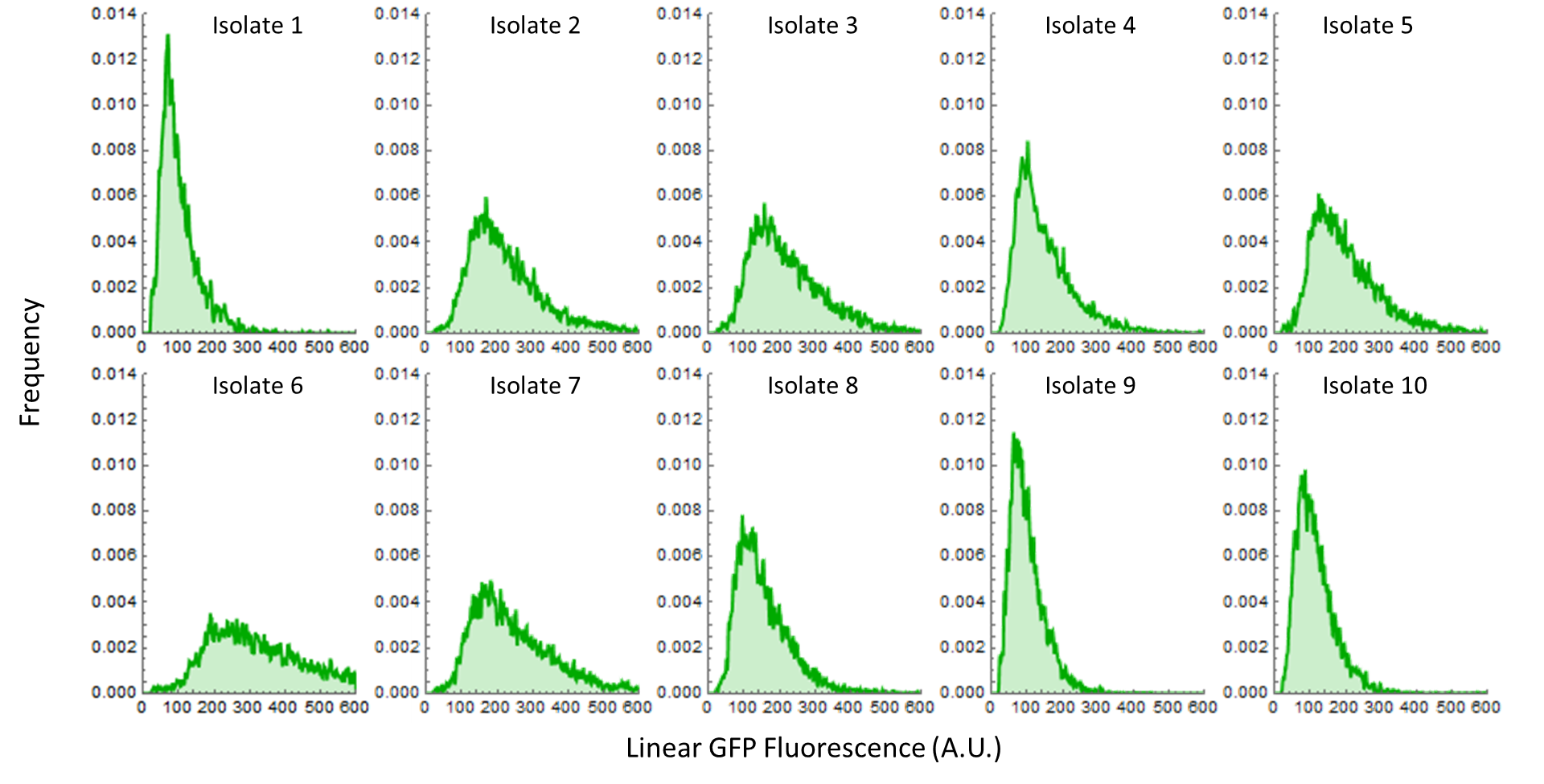
**

**Supplementary Figure 1.** GFP fluorescence distribution of 10 isolated library members. Ten isolates from the synonymously mutated library were measured with flow-cytometry to provide data to validate the sort-seq method. Some library isolates have low mean GFP fluorescence (e.g. Isolate 1) while others have high mean GFP fluorescence (e.g. Isolate 6).


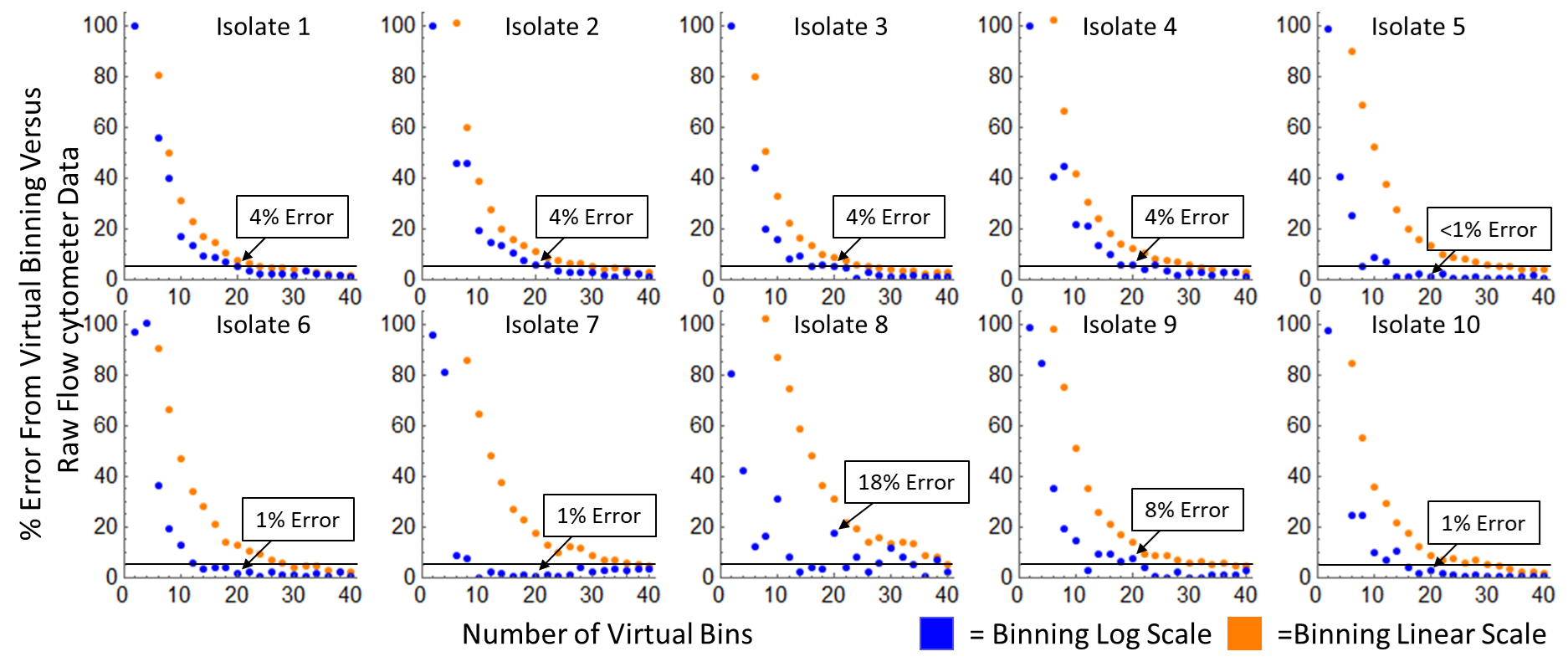


**Supplementary Figure 2.** Virtual binning of 10 isolates from the library. Ten isolates from the library were measured with flow-cytometry and the data was virtually binned on either linear (yellow) or log (blue) fluorescence scales to determine which method should be used for sort-Seq. Different number of virtual bins was used. The percent error was calculated by comparing the CV^2^_binned_ to CV^2^_real_ measured from flow-cytometer. The black line in each plot represents 5% error.


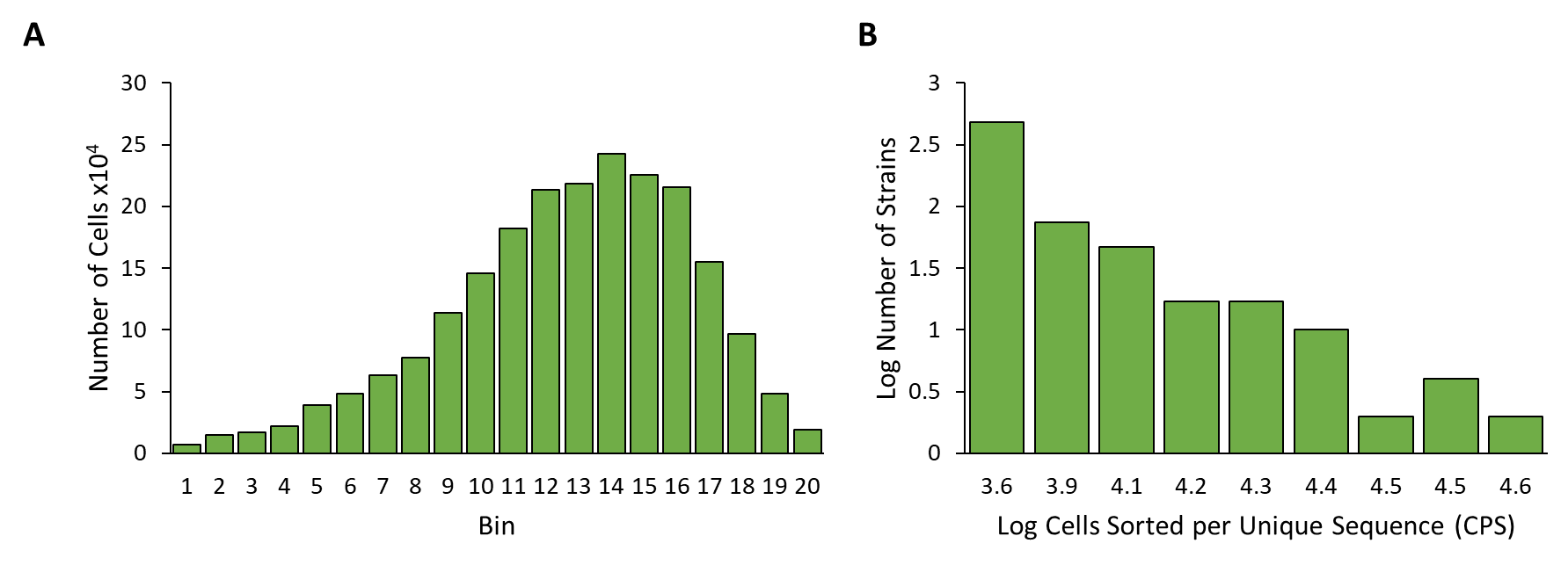


**Supplementary Figure 3**. Sorting cells based on GFP fluorescence. Results for each of the bins from one of the sort-seq experiments. (A) The number of cells sorted into each of the bins during FACS. A total of 2.16 million cells were sorted. (B) After sequencing each bin, the number of reads within each bin was calculated. High-throughput sequencing produced a total of over 2 million reads.


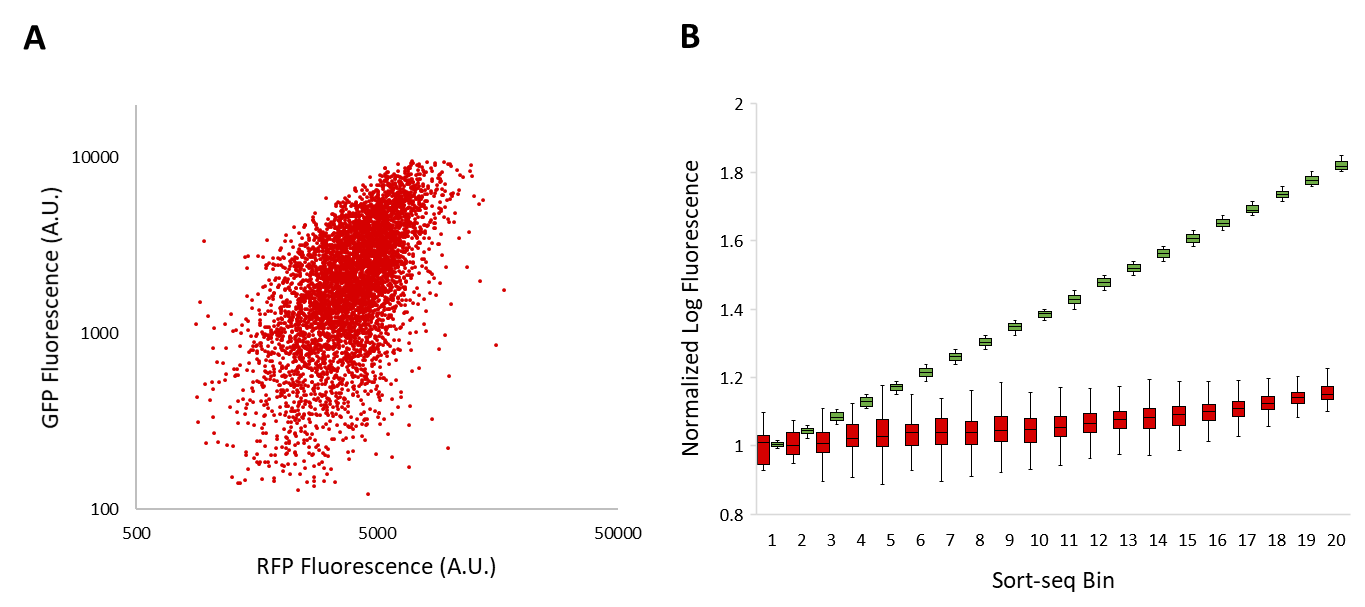


**Supplementary Figure 4.** Sort-seq RFP fluorescence. (A) The distribution of RFP fluorescence compared to GFP fluorescence of a sample (N = 5135) of the population used in the Sort-seq experiment. (B) The RFP (red) and GFP (green) distributions falling within each Sort-seq bin for a sample population (N= 5135) with error bars representing standard deviation.


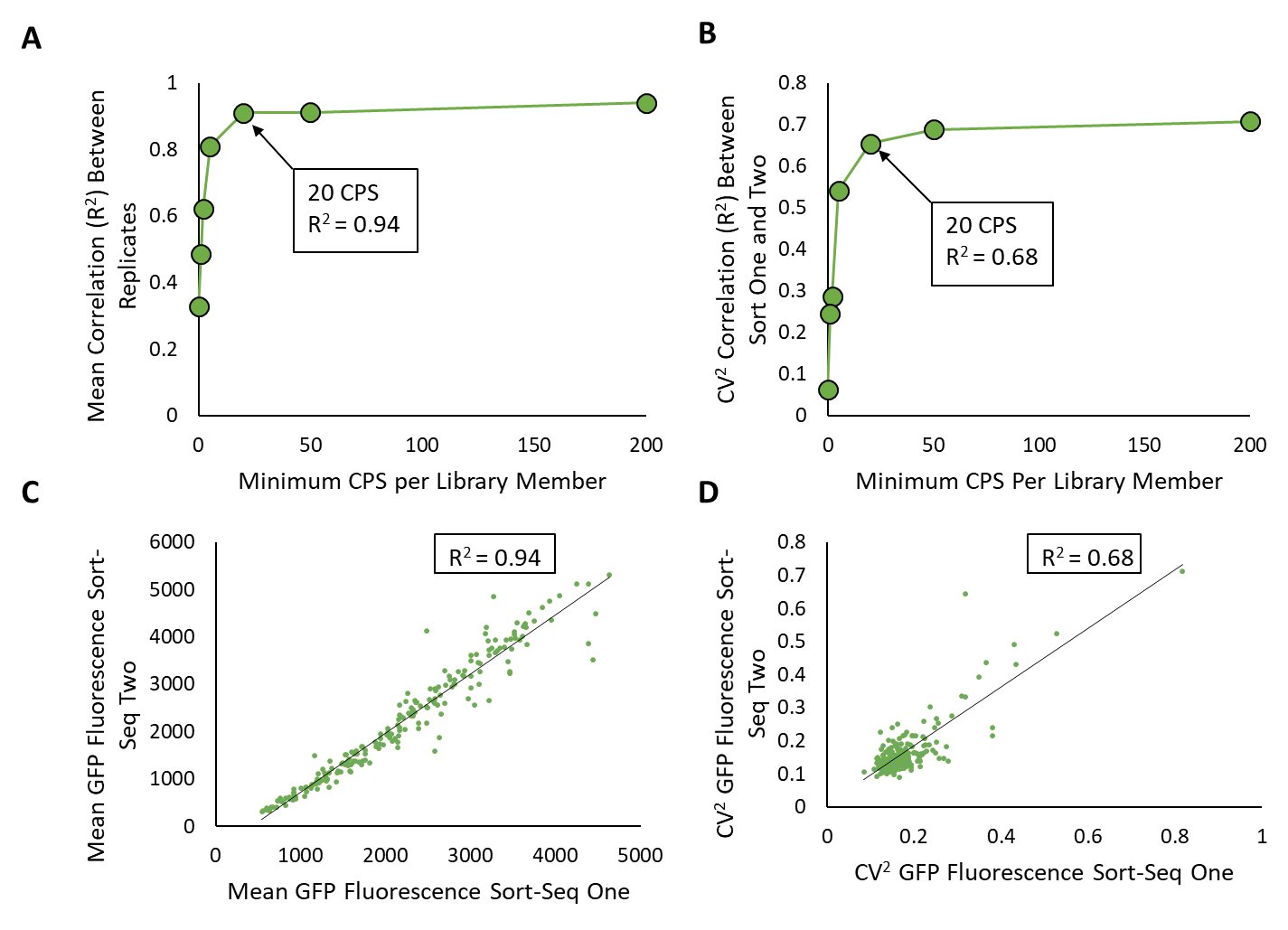


**Supplementary Figure 5.** Finding the minimum number of cell to use per sequence (CPS). Different CPS values were used as cut-off. Selected cells with higher CPS values were used to calculate GFP mean (A) and CV^2^ (B). (C) The correlation between two separate sort-seq experiments on mean GFP fluorescence at a CPS of 20. (D) The correlation between two separate sort-seq experiments on GFP CV^2^ at a CPS of 20.


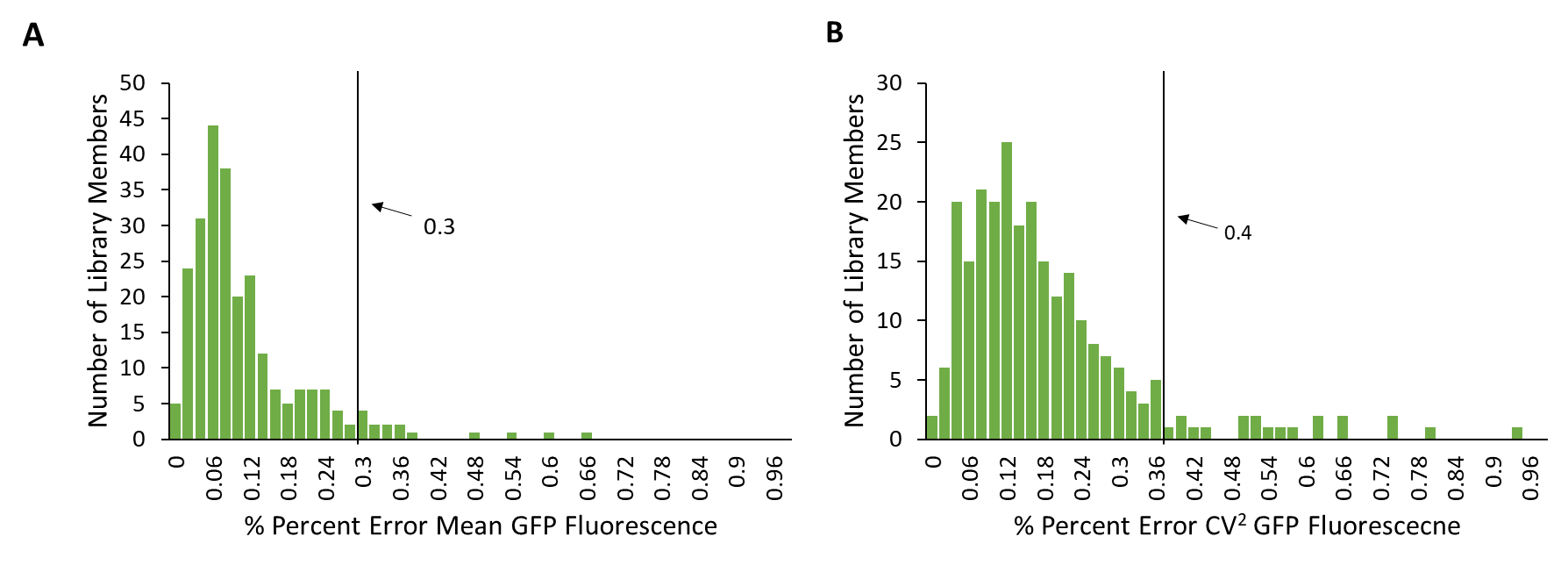


**Supplementary Figure 6.** Percent Error between three sort-seq experiments. Using all three sort-seq experiments, percent error is calculated in the measurement of both mean GFP fluorescence and CV^2^. (A) The percent error in mean GFP fluorescence across three different sort-seq experiments. Any library members above 30% error are excluded from further analysis. (B) The percent error in CV^2^ of GFP fluorescence across three sort-seq experiments. Any library members above 40% error are excluded from further analysis.


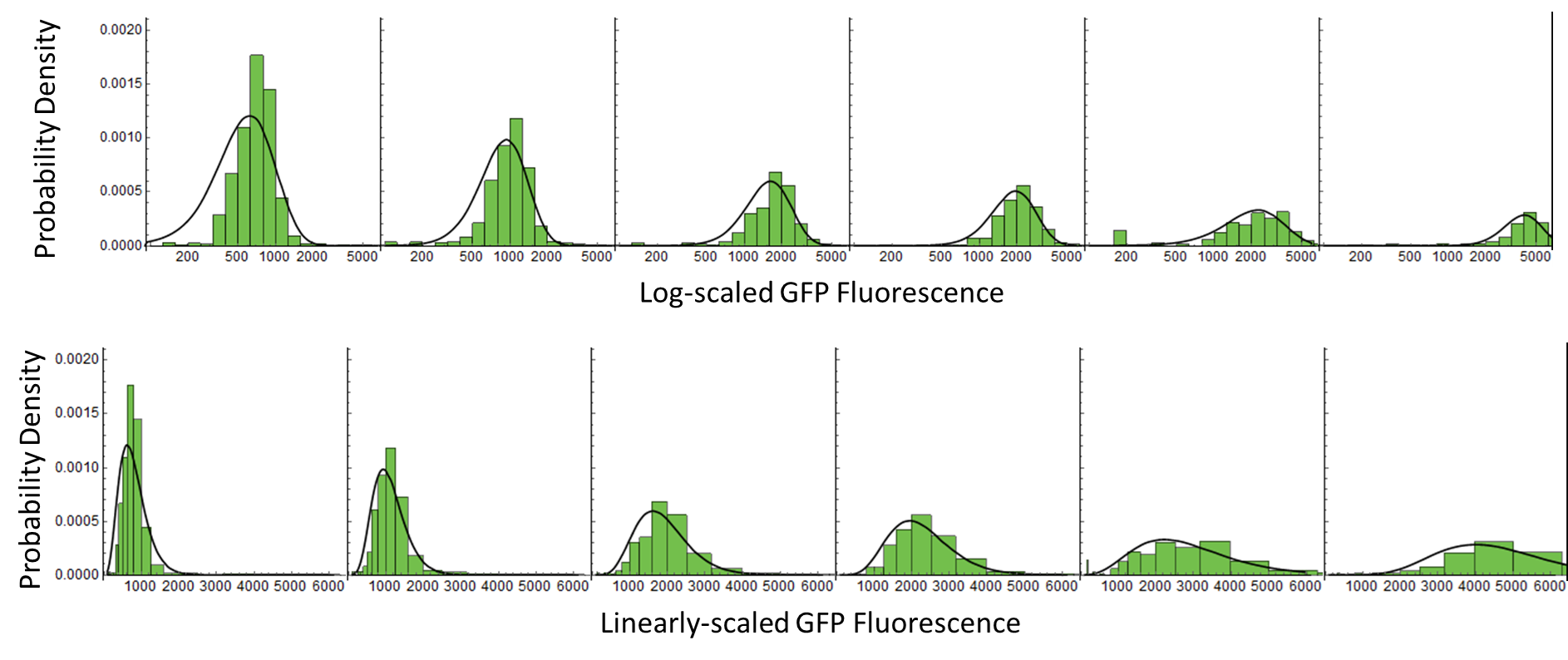


**Supplementary Figure 7.** Sort-seq-reconstructed single cell fluorescence (green columns) and the fitted curves (black) to a Gamma distribution for six library isolates as shown in Figure 2. The six isolates are plotted on both Log-scaled and Linearly-scald GFP fluorescence to emphasize the fitting of the Gamma distribution.


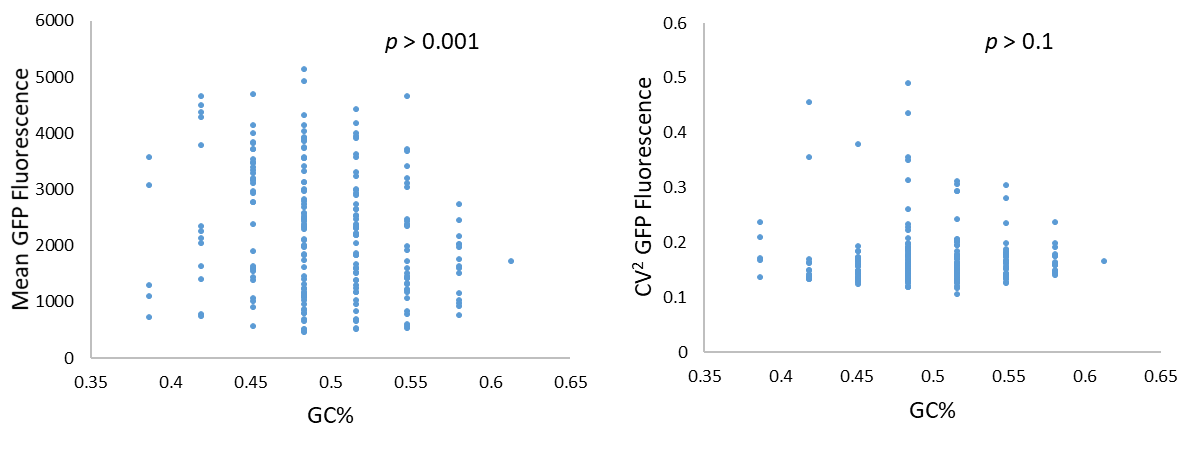


**Supplementary Figure 8.** The GC percent content of the synonymously mutated sequence is compared to the mean and CV^2^ GFP fluorescence of each sequence. The mean and CV^2^ GFP fluorescence is not affected by the GC content.
